# Patterns and trends of contraceptive use among sexually active adolescents in Burkina Faso, Ethiopia, and Nigeria: evidence from cross-sectional studies

**DOI:** 10.3402/gha.v8.29737

**Published:** 2015-11-09

**Authors:** Sennen Hounton, Aluisio J. D. Barros, Agbessi Amouzou, Solomon Shiferaw, Abdoulaye Maïga, Akanni Akinyemi, Howard Friedman, Desmond Koroma

**Affiliations:** 1 United Nations Population Fund, New York, NY, USA; 2 International Center for Equity in Health, Federal University of Pelotas, Capão do Leão, Brazil; 3 UNICEF, New York, NY, USA; 4 Department of Reproductive Health and Health Service Management, School of Public Health, Addis Ababa University, Addis Ababa, Ethiopia; 5 Centre for Demographic Research, Université Catholique de Louvain, Louvain-la-Neuve, Belgium; 6 Institut Supérieur des Sciences de la Population, Ouagadougou University, Ouagadougou, Burkina Faso; 7 Department of Demography and Social Statistics, Obafemi Awolowo University, Ile-Ife, Nigeria

**Keywords:** adolescents, childbearing, modern contraception, sub-Saharan Africa

## Abstract

**Background:**

The benefits of universal access to voluntary contraception have been widely documented in terms of maternal and newborn survival, women's empowerment, and human capital. Given population dynamics, the choices and opportunities adolescents have in terms of access to sexual and reproductive health information and services could significantly affect the burden of diseases and nations’ human capital.

**Objectives:**

The objectives of this paper are to assess the patterns and trends of modern contraception use among sexually active adolescents by socio-economic characteristics and by birth spacing and parity; to explore predictors of use of modern contraception in relation to the health system; and to discuss implications of the findings for family planning policy and programmes.

**Design:**

Data are from the last three Demographic and Health Surveys of Ethiopia, Burkina Faso, and Nigeria. The descriptive analysis focused on sexually active adolescents (15- to 19-year age group), used modern contraception as the dependent variable, and a series of contact points with the health system (antenatal care, institutional delivery, postnatal care, immunisation) as covariates. The multivariate analysis used the same covariates, adjusting for socio-economic variables.

**Results:**

There are two different groups of sexually active adolescents: those married or in a union with very low use of modern contraception and lower socio-economic status, and those unmarried, among whom nearly 50% are using modern contraception. Younger adolescents have lower modern contraceptive prevalence. There are significant inequality issues in modern contraception use by education, residence, and wealth quintile. However, while there was no significant progress in Burkina Faso and Nigeria, the data in Ethiopia point to a significant and systematic reduction of inequalities. The narrowing of the equity gap was most notable for childbearing adolescents with no education or living in rural areas. In the three countries, after adjusting for socio-economic variables, the strongest factors affecting modern contraception use among childbearing adolescents were marriage and child immunisation.

**Conclusions:**

Addressing child marriage and adopting effective policies and strategies to reach married adolescents are critical for improving empowerment and human capital of adolescent girls. The reduction of the equity gap in coverage in Ethiopia warrants further studies and documentation. The results suggest a missed opportunity for maternal and newborn and family planning integration.

**Paper context**

The choices and opportunities adolescents have in terms of access to sexual and reproductive health information and services could significantly impact nations’ human capital. We assessed patterns and trends of modern contraception use among sexually active adolescents in three countries (Burkina Faso, Ethiopia, and Nigeria) and explored health system predictors. The strongest predictors of contraception use among sexually active adolescents were marriage and child immunisation. There were pervasive inequality issues in contraception use by education, residence, and wealth quintile among sexually active adolescents; however, in Ethiopia, the data point to a significant and systematic reduction of inequalities, warranting further studies.

In the last few years, there has been a renewed effort from the global community to deliver contraception information and services to millions of women and adolescent girls who need them. The effects of family planning on maternal, perinatal, infant, and child health have been established through a systematic review of causal mechanisms (1). Population dynamics are central to sustainable development, and adolescents (defined here as 15–19 years old) are one of the fastest growing cohorts. Thus, understanding the patterns of contraception use among adolescents at the national and subnational levels could assist in designing strategies and programmes to better address coverage, quality, and equity issues at the country level (2–5). According to the 2014 State of the World Population Report (6), the world population includes the largest cohort of adolescents ever. The choices and opportunities that this adolescent group will have in terms of sexual and reproductive health information and services will determine, for example, the burden of adolescent pregnancies, unwanted pregnancies, abortions, HIV infections, and high school dropouts and may affect the ability of countries to harness the demographic dividend. Moreover, preventing adolescents from accessing comprehensive sexual and reproductive health information and services including family planning is a violation of their rights. Effectively addressing these burdens requires a better understanding of current patterns and trends of contraceptive use.

This paper examines data from Demographic and Health Surveys (DHS) in Burkina Faso, Ethiopia, and Nigeria. These countries were selected because of availability of survey data as part of the latest DHS rounds and because they provide a mix of characteristics, including geography, culture, religion, political decentralisation, population size, contraceptive prevalence, language, region, and economic indicators, representing some of the diversity in sub-Saharan Africa. In this paper, we assess sexually active adolescents in terms of their current use of modern contraception and trends in use. We also explore some subgroups, so that we can build a comprehensive picture of the dynamics of adolescent contraception use in these countries.

## Data and methods

We used data from publicly available national surveys (DHS) of Burkina Faso, Ethiopia, and Nigeria where information on adolescents’ sexual activity and contraception use were available. Burkina Faso is a landlocked country in West Africa with a low average contraception use and ranks 181 on the 2014 Human Development Index. Nigeria is also in West Africa, the most populous country in Africa with over 180 million inhabitants, a very low average contraceptive prevalence, and ranking 152 on the 2014 Human Development Index. Ethiopia is located in the Horn of Africa, with an estimated population of 80 million inhabitants, a federal government system similar to that in Nigeria, a recent increase in modern contraception use, and a ranking of 173 on the 2014 Human Development Index. Two surveys were included from Burkina Faso (2003 and 2010), three from Ethiopia (2000, 2005, and 2011), and three from Nigeria (2003, 2008, and 2013). The analyses focused on the use of modern methods of contraception among sexually active adolescents. Descriptive statistics on demographic factors (age and marriage), socio-economic factors (education, location, and wealth quintile), and birth risks (parity and birth spacing) were presented. Age was disaggregated into two groups (15–17 years and 18–19 years) to assess any differences for younger adolescents. Geographic differential effects were assessed at both urban and rural levels and also by states and regions. *Short spacing* was defined for women with at least two births as within a period of less than 24 months; parity was categorised as either zero, one, two, or more than two; and education was divided into no education, primary school completion, secondary level, or higher than secondary. Wealth quintiles were computed using household asset ownership and principal component analysis as described by Filmer and Pritchett (7).

We explored the determinants of modern contraception use among adolescents using the latest DHS in a multivariate analysis, with special interest in the effect of contact with the health system. The indicators we used as proxies for contact with the system included the number of antenatal care (ANC) visits, institutional delivery (yes or no), a postnatal care visit for the mother in the 2 months following delivery (yes or no), child immunisation (three doses of DTP3 used as a proxy and categorised as yes or no), visit to the household by a family planning health worker (yes or no), visit to a health facility by the mother in the past 12 months (yes or no), and whether or not information and counselling on family planning was received during a visit to a health facility. For this analysis, a logistic regression was used, adjusting for the potential confounding effect of socio-economic characteristics (education, residence, wealth quintiles). All the analyses were performed with Stata 13.0 statistical software (8), taking into account the design characteristics of the surveys. Ethical clearances were secured by the organisations that carried out the original surveys.

## Results

The cumulative sample sizes were 17,087 for Burkina Faso, 16,515 for Ethiopia, and 38,948 for Nigeria.

### Distribution of modern contraception use among sexually active adolescents over time, by marital status and age

**Fig. 1:**
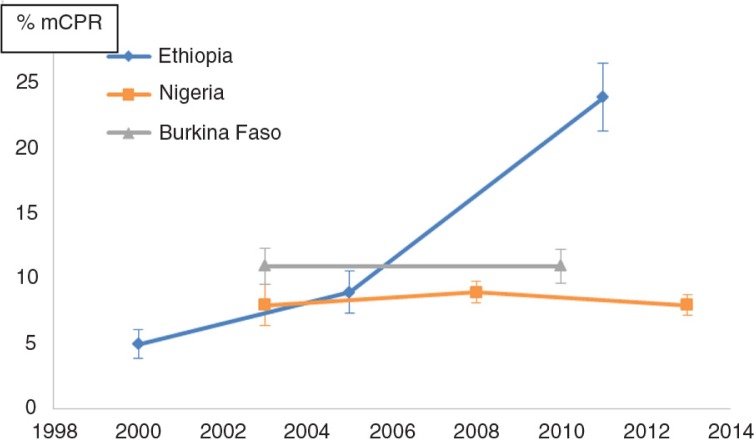
Trends in modern contraceptive use (% mCPR) among sexually active adolescents in Burkina Faso, Ethiopia, and Nigeria. Source: Demographic and Health Surveys.

**Fig. 2:**
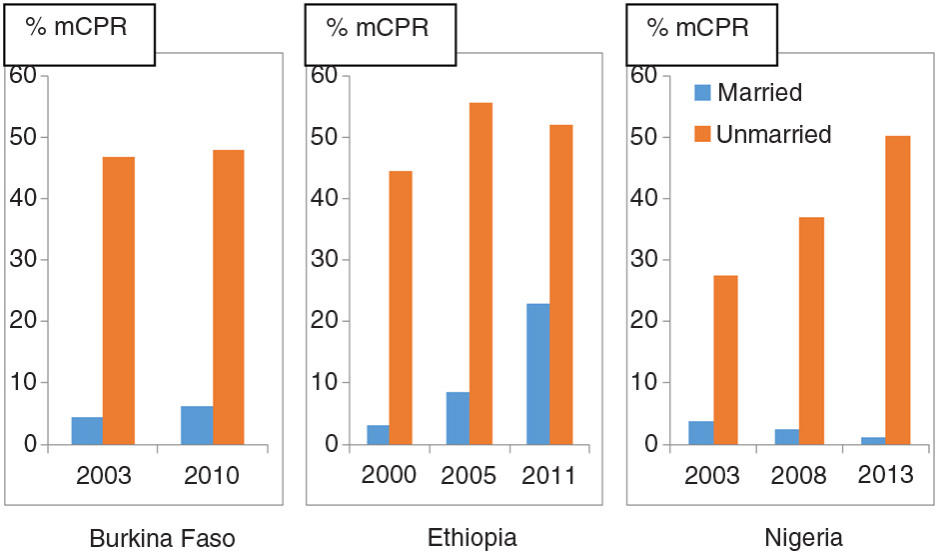
Trends in modern contraception use (% mCPR) among sexually active adolescents by marital status in Burkina Faso, Ethiopia, and Nigeria. Source: Demographic and Health Surveys.

Using the latest available DHS, most sexually active adolescents in the three countries studied were either married or in a union (henceforth referred to as *in union*): 88% (*N*=1,127), 96% (*N*=846), and 86% (*N*=2,480) in Burkina Faso, Ethiopia, and Nigeria, respectively. The prevalence of modern contraception use among sexually active adolescents was low in the three countries, the latest estimates being 11.2% for Burkina Faso, 24.1% for Ethiopia, and 7.8% for Nigeria (Fig. 1). There was a substantial difference in terms of modern contraception use between adolescents in union and those who were not in union (Fig. 2). The latest prevalence of use in the latter group was 7.8 times, 2.3 times, and 43.65 times higher than those in union in Burkina Faso, Ethiopia, and Nigeria, respectively. Overall, the proportion of modern contraception use among sexually active adolescents has remained almost unchanged (Fig. 1) in both Burkina Faso (at 11%, from 2003 to 2010) and in Nigeria (at around 8%, from 2008 to 2013). In contrast, in Ethiopia, the prevalence of use has been increasing sharply over the last decade, from 5 to 24%. Figure 2 shows that this increase has happened mostly among women in union (96% of sexually active adolescents). In Nigeria, we observed the opposite – a steep increase in contraceptive use among women not in union (14% of sexually active adolescents), with a decrease of use among those in union.

There was no statistically significant difference between the adolescents in the 15- to 17-year-old and the 18- to 19-year-old age groups in the use of modern methods of contraception at specific points in time (the last three iterations of the DHS).

### Disaggregation by socio-economic status

Table 1 presents the trends in modern contraceptive use among sexually active adolescents by education, place (rural or urban), and wealth quintiles in Burkina Faso, Ethiopia, and Nigeria. For Burkina Faso and Nigeria, there was a consistent disparity in the distribution of use of modern contraception among sexually active adolescents by education level, with the more educated adolescents more likely to be users compared to those adolescents who had not been to school. There was no statistically significant change in the prevalence of modern contraception use in Burkina Faso and Nigeria. This is in contrast to Ethiopia, where there has been a large and positive average annual rate of change over the years across all education levels. In all three countries, there was a huge gap in use between those with primary-level education and all levels above. The latest prevalence of use among adolescents with a secondary-level education or above was 2.4 times, 1.9 times, and 5.9 times higher in Burkina Faso, Ethiopia, and Nigeria, respectively, than those who had completed only primary-level education.

**Table 1:** Trends in modern contraception use among sexually active adolescents in Burkina Faso, Ethiopia, and Nigeria, by education, geographic location, and wealth quintile

	Burkina Faso			Ethiopia				Nigeria			
			
Variables	2003	2010	AAR*	2000	2005	2011	AAR*	2003	2008	2013	AAR*
Education											
None	7.4	5.5	−2.1	3.1	5.4	14.2	10.1	1.0	0.8	0.2	−12.6
Primary	19.9	18.0	−0.7	12.7	14.1	28.8	5.6	8.6	5.6	4.6	−4.5
Secondary or greater	52.1	43.6	−1.3	9.8	59.0	54.6	11.3	27.8	27.3	27.0	−0.2
Place											
Urban	38.4	35.3	−0.6	18.7	46.7	49.0	6.5	11.7	20.6	22.9	4.6
Rural	5.8	5.1	−0.9	3.4	6.6	20.9	11.9	6.6	6.1	4.0	−3.6
Wealth quintiles											
Lowest	4.1	2.9	−2.5	3.2	3.4	16.7	11.0	3.3	1.8	0.3	−19.0
Second	4.1	4.5	0.6	1.0	4.7	18.4	18.2	2.1	5.0	2.6	1.3
Middle	6.5	4.2	−3.2	1.0	3.2	19.9	19.1	4.4	8.5	10.4	5.8
Fourth	10.7	10.0	−0.5	0.8	11.9	24.3	21.0	16.5	18.7	19.5	1.2
Highest	34.2	36.7	0.5	20.9	31.0	52.3	6.2	20.9	36.1	39.2	4.3

AAR*: average annual percent rate of change between the latest and earliest DHS in the series considered for the analysis; none: no education; primary: primary completed; secondary or greater: above primary school.

Residence follows a pattern similar to education, with a greater gap between urban and rural areas. The latest prevalence of use among adolescents in urban areas was almost 7 times, 2.3 times, and 5.7 times greater than those who lived in rural areas in Burkina Faso, Ethiopia, and Nigeria, respectively. In addition, in Ethiopia, it is important to note an almost 2.6-fold and 6.1-fold increase in modern contraception use by adolescents from 2000 to 2011, in urban and rural areas, respectively. Moreover, the equity gap between urban and rural areas has been decreasing over time, and a similar pattern was observed to some extent in the differences in educational level and wealth quintile. Systematic analyses of the reasons for success in reduction in equity gaps will be important in order to understand the results and share lessons with other countries with prevailing inequities.

### Subnational variations in modern contraception use among sexually active adolescents in Burkina Faso (2010), Ethiopia (2011), and Nigeria (2013)

**Fig. 3:**
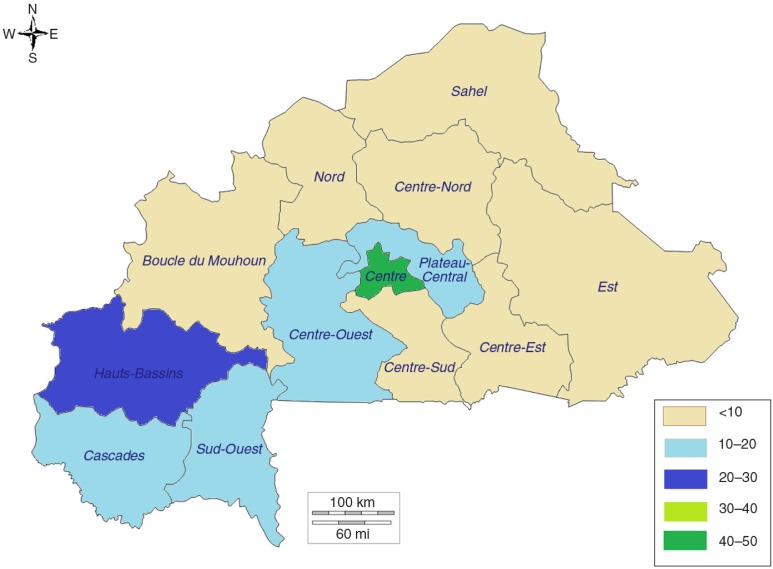
Mapping of prevalence of modern contraception use among sexually active adolescents by region, Burkina Faso. Source: 2010 Demographic and Health Survey.

**Fig. 4:**
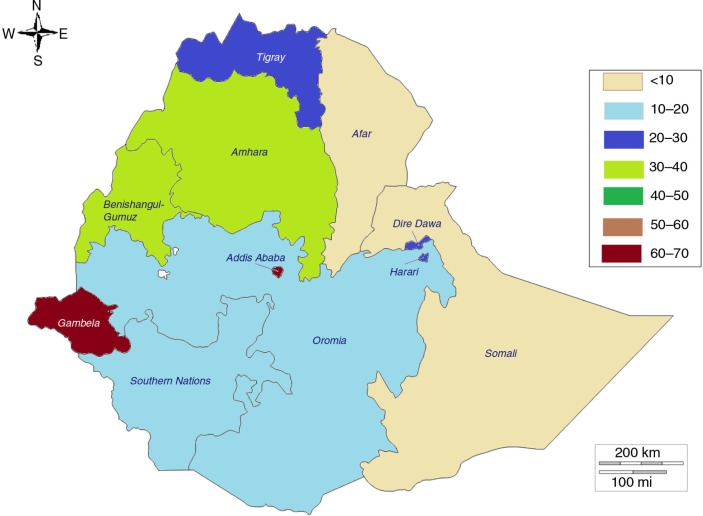
Mapping of prevalence of modern contraception use among sexually active adolescents by region, Ethiopia. Source: 2011 Demographic and Health Survey.

**Fig. 5:**
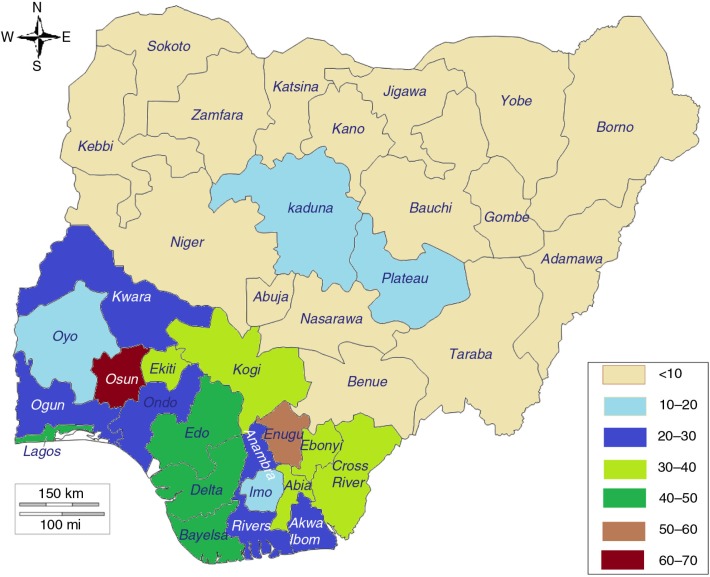
Mapping of prevalence of modern contraception use among sexually active adolescents by state, Nigeria. Source: 2013 Demographic and Health Survey.

As reflected by the distribution of modern contraception use among adolescents in rural and urban areas, there were large differences in the proportions of sexually active adolescents using modern contraception in the three countries (Figs. 3–5). As reflected in the differentials between urban and rural areas, the highest prevalence of use of modern contraception was recorded in the regions hosting the two main cities in Burkina Faso (Centre and Hauts-Bassins); the Addis Ababa region in Ethiopia; and Osun, Enugu, and Lagos States in Nigeria.

There were similarities across the three countries in the effect of region: the lowest percentages of use were recorded in predominantly Islamic regions and states (the Sahel, Nord, and Centre-Nord regions in Burkina Faso; the Afar and Somali regions in Ethiopia; and northern Nigeria). In Nigeria, it was striking to see almost no use of modern contraception among sexually active adolescent girls in Sokoto, Yobe, Zamfara, Kano, Katsina, Kebbi, Jigawa, Nasarawa, Borno, and Bauchi, which are all in northern Nigeria. Although there is homogeneity in northern Nigeria, significant differences between states could be observed within other regions. For example, although the average modern contraception use estimate among sexually active adolescents in the south-east in 2013 was 32.6%, Enugu State recorded 50.7% while Imo State recorded 13.2%. Similarly, although the use estimate among sexually active adolescents in the south-west in 2013 was 33.9%, Osun State recorded 65% (95% CI: 45–81%), while Oyo State recorded 15% (95% CI: 4.8–38%).

### Distribution of modern contraception use by parity and birth spacing

In the latest DHS, the proportions of sexually active adolescents without a child, with one child, and with two or more children was 53.2, 39.8, and 7% in Burkina Faso, 55.3, 33.6, and 11.1% in Ethiopia, and 54.8, 35, and 10.2% in Nigeria, respectively. The effect of parity on the pattern of modern contraception use among sexually active adolescents over the years was similar in Burkina Faso and Nigeria (Table 2). There was no significant trend in modern contraception use among sexually active adolescents with one child and two or more children as compared to those with no children. In Ethiopia, however, there was an increasing trend in modern contraception use over the years, irrespective of parity. Modern contraception use had increased among first-time young mothers from 12% in 2005 to 24% in 2011.

**Table 2:** Patterns of modern contraception use by parity and marital status among sexually active adolescents in Burkina Faso, Ethiopia, and Nigeria

Countries	Burkina Faso, 2010 DHS		Ethiopia, 2011 DHS		Nigeria, 2013 DHS	
			
Covariates	In union	Not in union	In union	Not in union	In union	Not in union
Parity	Prevalence	Prevalence	Prevalence	Prevalence	Prevalence	Prevalence
0	6.2	47.3	23.7	56	0.1	51.8
(95% CI)	3.3–11.2	37–57.8	17.4–31.6	32.8–76.8	0.0–0.4	45.3–58.3
1	5.7	65.3	24	72	2.2	33.1
(95% CI)	3.7–8.5	21.8–92.7	17.3–32.2	16.2–97.2	1.3–3.6	18.7–51.6
2 or more	9.0	0	15	0	1.9	43.3
(95% CI)	4.5–17.3	−	7.3–29.7	–	0.6–5.5	5.9–88.8

CI: confidence interval; DHS: Demographic and Health Survey. Missing values are due to very small sample sizes.

The relationship between modern contraception use and parity varied significantly by marital status. Using the latest DHS in each of the three countries, the prevalence of modern contraception use among sexually active adolescents with one child, who were not in union, was 65.3, 72.2, and 33.1% in Burkina Faso, Ethiopia, and Nigeria, respectively. These are 11.5 times, 3.0 times, and 15.3 times the prevalence for those in union in these countries, respectively.

Similarly, for adolescents with no child, prevalence of modern contraception use among adolescents not in union was 47.3, 56, and 41.3%, which was 7.7 times, 2.4 times, and 411 times the prevalence for those in union in Burkina Faso, Ethiopia, and Nigeria, respectively. These differences across countries are staggering and may point to a strong negative effect of marriage on the ability of adolescent girls to control their own fertility, irrespective of the level of modern contraceptive prevalence. It is also possible that, once married, adolescents want to conform to social norms and do not want to use contraceptives, although the data on unmet need for modern contraception among adolescents support the latter hypothesis less (6).

Regarding birth spacing, there was an inherent limitation for adolescents 15–19 years in the data sets for the analysis in terms of sample size, given that birth spacing can only be reliably explored for mothers with at least one child and a lag time of 24 months after each birth. For example, if an adolescent girl has a child at age 18, we do not have enough lag time in our sampling frame (limited to her 20th birthday) to observe whether or not she would have had short birth spacing or normal birth spacing as she moves out of the adolescent age group. For the purpose of this analysis, we decided to look mainly at any differences in the prevalence of modern contraception use among short birth spacing versus spacing of at least 24 months (normal spacing). There was no significant difference in the prevalence of modern contraception use by adolescent mothers by birth interval (spacing). The very small sample sizes after disaggregation did not allow for a comparative analysis of spacing by marital status.

### Effects of selected health services on modern contraception use among sexually active adolescents

We explored the effects on the use of modern contraception of ANC visits, institutional delivery, a postnatal care visit in the 2 months following delivery, child immunisation (DTP3), visit to the household by a family planning health worker, visit to a health facility by the mother in the past 12 months, and receipt of information and counselling on family planning during a visit to a health facility. We explored the variables individually and in a model, for all sexually active adolescents as well as for childbearing adolescents. The overall results underscore that, regardless of modern contraceptive prevalence rate, more contacts with health services are associated with increased modern contraception use.

More specifically, in Burkina Faso, the prevalence of modern contraception use among sexually active adolescents was 9.2% for four ANC visits, 7.9% for childbearing adolescents with postnatal care for the baby, 10.3% for adolescents whose babies completed DTP3, and 8.6% for adolescents who delivered in health facilities. These were approximatively 1.5 times, 13 times, and 7.3 times the prevalence for those with only one ANC visit, no postnatal care visit, no DTP3, and no institutional delivery, respectively. We did not observe a significant effect of household visit by a family planning health worker, visit to a health facility by the mother in the past 12 months, or receipt of information and counselling on family planning during a visit to a health facility.

In Ethiopia, there was a positive association of contraceptive use with all of the variables. The prevalence of modern contraception use among sexually active adolescents was 51.6% for four ANC visits, 48.5% for childbearing adolescents who used postnatal care services, 43.3% for adolescents whose babies completed DTP3, 22.1% for adolescents who delivered in health facilities, 37.6% for those who had a household visit by a family planning health worker, 37.4% for those who had visited a health facility in the past 12 months, and 37.5% for those who received information and counselling on family planning during a visit to a health facility. These values were approximately 3.6, 2.5, 2.5, 1.1, 1.6, 2, and 16.3 times the prevalence for those with only one ANC visit, no postnatal care visit, no DTP3, no institutional delivery, no visit by family planning health worker, no visit to a health facility in the past 12 months, and no counselling on family planning during a visit to a health facility, respectively.

In Nigeria, the pattern was very similar to that observed in Burkina Faso, with a positive association of use with four ANC visits, postnatal care for the baby, completion of DTP3, and institutional delivery but no effect of household visit by a family planning health worker, visit to a health facility by the mother in the past 12 months, or receipt of information and counselling on family planning during a visit to a health facility. Subsequently, we elected to assess variance in modern contraception use among sexually active adolescents that could be explained by opportunistic contacts with the health system, such as ANC, institutional delivery, and child immunisation, adjusting for socio-economic variables (education, residence, and wealth quintile) and marital status. The results are presented in Table 3.

**Table 3:** Univariate and multivariate logistic regression of the effect of health service contacts on modern contraception use among adolescents in Burkina Faso, Ethiopia, and Nigeria

Variables	Crude OR (95% CI)	Adjusted OR (95% CI)
Burkina Faso		
Antenatal care (4 visits)	1.6 (0.8–3.2)	1.1 (0.5–2.4)
Child immunisation^a^ (completion of DTP3)	8.0 (1.8–35.9)	8.9 (3.0–26.7)
Institutional delivery (yes)	8.5 (1.1–63.2)	2.9 (0.3–26.3)
Marital status (married)	0.07 (0.04–0.11)	0.2 (0.0–2.4)
Education		
Primary	3.8 (2.2–6.5)	0.9 (0.3–2.8)
Secondary or above	13.3 (7.9–22.5)	1.2 (0.3–3.4)
Residence (urban)^a^	10.2 (6.6–15.7)	3.7 (1.5–9.3)
Wealth quintiles		
Second	1.6 (0.6–4.1)	2.4 (0.3–20.6)
Middle	1.5 (0.5–3.9)	1.6 (0.2–15.2)
Fourth	3.7 (1.5–9.4)	3.4 (0.4–31.3)
Highest	19.4 (8.2–4.6)	6.7 (0.8–56.7)
Ethiopia		
Antenatal care		
1 visit	0.6 (0.2–1.4)	0.9 (0.3–2.4)
4 visits	3.7 (1.3–10.3)	2.8 (0.9–8.7)
Child immunisation^a^ (completion of DTP3)	3.7 (1.6–8.4)	3.5 (1.4–8.3)
Institutional delivery (yes)	2.1 (0.7–6.1)	4.9 (0.4–52.7)
Marital status (married)^a^	0.3 (0.1–0.6)	0.02 (0.00–0.74)
Education		
Primary^a^	2.5 (1.5–4.0)	3.1 (1.1–8.5)
Secondary or above	7.3 (2.8–19.2)	4.6 (0.4–52.7)
Residence (urban)	3.6 (1.8–7.2)	10.3 (0.6–164.7)
Wealth quintiles		
Second	1.1 (0.5–2.4)	0.5 (0.2–1.9)
Middle	1.2 (0.6–2.6)	0.5 (0.2–1.6)
Fourth	1.6 (0.8–11.6)	0.4 (0.1–2.0)
Highest	5.5 (2.6–11.6)	1.3 (0.2–11.8)
Nigeria		
Antenatal care		
1 Visit	0.5 (0.1–2.5)	1.4 (0.3–7.8)
4 Visits	4.3 (1.1–16.8)	2.4 (0.6–9.5)
Child immunisation (completion of DTP3)	8.6 (3.8–19.2)	1.9 (0.7–5.0)
Institutional delivery (yes)	3.5 (1.6–7.7)	0.7 (0.3–1.7)
Marital status (married)^a^	0.01 (0.00–0.02)	0.1 (0.03–0.40)
Education		
Primary	25.5 (5.5–128.8)	4.3 (0.7–27.8)
Secondary or above^a^	196.8 (45.5–851.5)	7.7 (1.5–40.5)
Residence (urban)	7.1 (5.1–9.9)	1.1 (0.4–2.7)
Wealth quintiles		
Second	9.2 (2.8–30.9)	2.4 (0.3–18.5)
Middle	40.5 (12.7–129.3)	2.5 (0.4–14.2)
Fourth	85.1 (26.8–270.3)	5.3 (0.9–31.5)
Highest	226.0 (70.1–728.5)	2.6 (0.3–22.4)

a Significant at the 5% level. Crude and adjusted odds ratio (OR) estimates with their 95% confidence interval (95% CI) of the effect of selected health services on modern contraception use among sexually active adolescents in Burkina Faso (2010 DHS), Ethiopia (2011 DHS), and Nigeria (2013 DHS). Crude OR calculated from a univariate logistic regression. Adjusted OR calculated from a multivariate logistic regression adjusting for other variables in the model.

In a simple, non-adjusted multivariate analysis, there were several variables that were associated with use of modern contraception among sexually active adolescents in all three countries. These included child immunisation, marital status, education (all levels), residence, and wealth quintile (least poor). After adjusting for socio-economic variables, there were fewer variables with a statistically significant association with modern contraceptive use among sexually active adolescents. These included child immunisation and residence in Burkina Faso; child immunisation, marital status, and education (primary level) in Ethiopia; and marital status and education (secondary level and above) in Nigeria.

## Discussion

### Demographic and socio-economic variations across countries

There were significant variations in the use of modern contraception by demographic and socio-economic characteristics in Burkina Faso, Ethiopia, and Nigeria, despite being in the same geographic region of sub-Saharan Africa. While in Burkina Faso and Nigeria there has been no significant progress over the last three surveys at the national level, the data from the 2011 DHS in Ethiopia indicate a sharp increase in the use of modern contraceptives among sexually active adolescents. It will be important to identify the policies and strategies being implemented in Ethiopia and share these findings with other countries. Regarding age, although the difference was not statistically significant, the use of modern contraception tended to be lower for younger adolescents. This finding is confirmed by a recent study in Kenya, Tanzania, and Uganda (9) where the authors found the relationship between adolescent motherhood and lack of contraception use was strongest among births within the youngest age group (<16 years old). For marital status, the main finding in our study, also confirmed by other authors (9, 10), is that adolescents in union constitute a vastly different group from adolescents not in union. One of our main questions was whether adolescent women in union would be more likely to use contraception after having their first child. The data in our study suggest that first-time, married young mothers are not using modern contraception. They are also more likely to be less educated, poorer, and to be in rural areas as compared to those that are not married. This situation increases their vulnerability for increased fertility and higher risks of mortality and fistula.

The data on socio-economic variables allow us to analyse equity issues. Across all three countries, there is a significant equity gap in modern contraception use. Adolescents who have a high school level education or above, who are in urban areas, and who are in the highest wealth quintiles use significantly more modern contraception as compared to their peers who have primary-level education, live in rural areas, or who belong to the lowest wealth quintiles. These findings are similar to the trends and patterns observed in sub-Saharan Africa or at the global level by other authors (11, 12). However, the countries differ in the direction and type of changes observed over time. In Nigeria, the equity gap has worsened over the years, with a reduction in the average annual rate in modern contraceptive prevalence of 12.6, 3.6, and 19% for childbearing adolescents with no education, living in rural areas, or belonging to the lowest wealth quintile, respectively. The lowest quintile is the only category within the wealth quintiles to have a huge increase in the equity gap as compared to the other quintiles. The data also suggest a similar pattern in Burkina Faso, where the worst increase in the equity gap affecting modern contraception use among childbearing adolescents was observed for the lowest (first), second, and third quintiles, as well as for the group with no education.

In contrast, data from Ethiopia revealed a significant and systematic reduction in the equity gap over the years in terms of education, geographic location, and wealth quintiles. The narrowing of the equity gap was notable for childbearing adolescents with no education or living in rural areas, with an average annual increase in the rate of modern contraception use of 10 and 12%, respectively. The narrowing of the equity gap was greatest for the second, third, and fourth quintiles, with an average of 20% annual increase in the rate of modern contraception use among all three groups combined. These results are worth noting, given that Ethiopia has been cited as one of the countries with the highest inequities in health services among many other sub-Saharan countries (13, 14). Ethiopia has made massive investments in primary healthcare and a national community health-worker programme (referred to as the *Health Extension Workers Programme*), with community workers on the government payroll who are trained, supervised, and equipped to provide modern contraception at the last mile, including implants (15). The data also suggest a need for further research to investigate if the same reduction in the equity gap is observed across other maternal and newborn interventions. Systematic analyses of the reasons for success in reducing inequity will be important to document and share with other countries with greater inequities.

### Subnational variations

With increasing decentralisation and empowerment of local governments at subnational levels (states, regions, and counties are increasingly setting priorities and budget allocations across development sectors), it is critical to provide disaggregated data for evidence-based policy making and programme design. The data show some similarities across the three countries, with the lowest modern contraception rates recorded among sexually active adolescents in the poorest regions and states and in rural areas. It is also interesting to note that across the three countries there is very low contraceptive prevalence in largely Islamic populations, although predominantly animist regions in Burkina Faso also had very low prevalence rates. It is not surprising that these states and regions with very low contraception use also have the highest prevalence of child marriage and lowest literacy rates. In fact, child marriage has shown to be associated with unintended pregnancy, low levels of contraceptive use, and limited use of maternal health services (16), which result in increased vulnerability for negative maternal outcomes.

These factors alone, however, do not account for all the variance in contraception use across states and regions. Even within the same geographic areas, we observed significant variations among neighbouring states, such as is the case in Nigeria for the states of Osun and Oyo in the south-west or between the states of Imo and Abia in the south-east. These differences point to possible differences in policy, strategies, and investment by local governments in women's, children's, and adolescents’ health, as well as in cultural and societal norms and values with regards to keeping adolescent girls in school, curtailing child marriage, and increasing access to modern contraception for all women of reproductive age.

### Determinants of modern contraceptive use among sexually active adolescents

Using the latest DHS data, the multivariate analysis of potential factors explaining the variance in modern contraception use among childbearing adolescents was very revealing and points to some commonalities, as well as missed opportunities, for integrating family planning services with maternal and newborn health across the three countries. There was a very strong and negative impact of marriage, even after adjusting for all other variables. The adjusted odds of a childbearing adolescent using modern contraception when she is not in union are 5 times, 50 times, and 10 times that of married adolescents for Burkina Faso, Ethiopia, and Nigeria, respectively. It is therefore critical to reach those childbearing adolescents in union in order to improve their maternal and newborn outcomes (16). Having at least a high school education, living in urban areas, and belonging to the highest wealth quintiles may just reflect the same group of adolescents who are better off as compared to others and who have more access to modern contraceptive information and services.

The pattern and strength of change in these factors also varies across countries after adjusting for socio-economic variables (education, residence, wealth quintiles). While in Burkina Faso the strongest associations with contraceptive use were observed for child immunisation and residence, in Ethiopia it was child immunisation, marital status, and education (primary school), and in Nigeria marital status and education (secondary school). Why ANC and institutional delivery did not significantly affect use of contraception after adjusting for socio-economic variables would be interesting to understand. A possibility is that these factors are influenced by residence in Burkina Faso, where health services are more accessible and available in urban areas; the same could be said for adolescents with primary-level education or above in Ethiopia and Nigeria, who may best represent those living in cities with more access to ANC and institutional delivery.

One can clearly see the importance of integration of maternal and newborn health services (17), particularly child immunisation, the strongest and most significant modifier of modern contraceptive use among childbearing adolescents. Are the childbearing adolescents more likely to become users of modern contraception when their children complete DTP3, or are the adolescents who ensure completion of their children's immunisation more likely to hear about contraception and services and adopt family planning?

Regardless, child immunisation appears to be a critical factor and thus a missed opportunity for improving the coverage of family planning among childbearing adolescents. The odds that a childbearing adolescent will use modern contraception when her newborn completes DTP3 (which corresponds roughly to a period of 3 months after birth) is 9 times, 3.5 times, and 2 times the odds in the absence of exposure to child immunisation in Burkina Faso, Ethiopia, and Nigeria, respectively. Finally, the results of the multivariate analysis, after adjusting for socio-economic and demographic factors, may hide the effects of other variables such as cultural norms. Policies and strategies to provide information and services for contraception, which are critical for the empowerment and rights of adolescent girls and women, need to build on the most contextually relevant drivers for use of modern contraception.

### Limitations

Some limitations of the study are related to the small sample sizes. Another limitation in the analysis of birth spacing is the short period available to observe birth spacing during adolescence, given the age restriction (15–19 years) and the definition of a normally spaced birth (24 months). These limitations often resulted in very wide confidence intervals, which limited statistical analysis and the conclusions that could be made.

## Conclusions

This paper highlights the importance of understanding subnational variations and differences in modern contraceptive use among sexually active adolescents in order to better address their needs. Although there are prevailing equity issues, the data from Ethiopia are an indication that, when proper policies and investments are made (e.g. the Health Extension Workers Programme and availability of modern contraceptives at the last mile) (15), it is possible to significantly reduce the equity gap in a short period of time. Marriage remains, by far, the major bottleneck for childbearing adolescents’ use of modern contraception. Improving modern contraception use among sexually active adolescents will require capitalising on missed opportunities for contact with the health system, particularly during child immunisation. Banning child marriage, ensuring comprehensive sexuality education and connecting childbearing adolescents with information and services during routine health services such as ANC, institutional delivery, and child immunisation are critical to meeting their needs and improving adolescent girls’ well-being, empowerment, and human capital.
